# Antibiotic resistance in probiotic bacteria

**DOI:** 10.3389/fmicb.2013.00202

**Published:** 2013-07-18

**Authors:** Miguel Gueimonde, Borja Sánchez, Clara G. de los Reyes-Gavilán, Abelardo Margolles

**Affiliations:** Instituto de Productos Lácteos de Asturias, Consejo Superior de Investigaciones CientíficasVillaviciosa, Spain

**Keywords:** probiotics, *Lactobacillus*, *Bifidobacterium*, *Bacillus*, antibiotic resistance

## Abstract

Probiotics are live microorganisms which when administered in adequate amounts confer a health benefit on the host. The main probiotic bacteria are strains belonging to the genera *Lactobacillus* and *Bifidobacterium*, although other representatives, such as *Bacillus* or *Escherichia coli* strains, have also been used. *Lactobacillus* and *Bifidobacterium* are two common inhabitants of the human intestinal microbiota. Also, some species are used in food fermentation processes as starters, or as adjunct cultures in the food industry. With some exceptions, antibiotic resistance in these beneficial microbes does not constitute a safety concern in itself, when mutations or intrinsic resistance mechanisms are responsible for the resistance phenotype. In fact, some probiotic strains with intrinsic antibiotic resistance could be useful for restoring the gut microbiota after antibiotic treatment. However, specific antibiotic resistance determinants carried on mobile genetic elements, such as tetracycline resistance genes, are often detected in the typical probiotic genera, and constitute a reservoir of resistance for potential food or gut pathogens, thus representing a serious safety issue.

## INTRODUCTION

One of the most important selection criteria for bacterial strains intended for use in the food industry is concern for their safety. In Europe, according to the Qualified Presumption of Safety (QPS) approach, established by the European Food Safety Authority (EFSA, 2008), the nature of any antibiotic resistance determinant present in a candidate microorganism should be determined prior to approval for QPS status. Therefore, antibiotic resistance *per se* is not a safety issue; it only becomes such when the risk of resistance transfer is present.

Those probiotics belonging to species included in the EFSA QPS list (EFSA, 2012) have excellent safety records, and detrimental effects produced as a consequence of their ingestion are very scarce (Gouriet et al., 2012). Undoubtedly, a full safety assessment begins with a proper identification of the strain and an *in vitro* evaluation of the potential risks. In this regard, the presence of antibiotic resistance determinants, and their potential mobility, deserves special attention. Currently, it is generally accepted that the possibility of transfer is related to the genetic basis of the resistance mechanism, i.e., whether the resistance is intrinsic, acquired as a result of a chromosomal mutation(s), or acquired by horizontal gene transfer.

Most probiotics are common members of the human intestinal tract, and they are ingested in large amounts in functional foods, and the presence of antibiotic resistance determinants in their genome must be systematically screened. For instance, the bifidobacterial population in the human gut can be as high as 10^11^ cells/g of intestinal content, and even if the presence of the resistance genes are not a threat when they are present in bifidobacterial cells due to their lack of infectivity, these cells can constitute a reservoir from which genes could be transmitted to pathogenic bacteria. Thus, it is of great interest to investigate whether these determinants can be transferred in the food and gut environment (Lahtinen et al., 2009). Furthermore, an important point to bear in mind is that animal probiotics are a source of live bacteria in the food chain, and in the European Union there has been an active policy to eliminate transmissible resistances in these products. Such concern must be also expressed regarding consumption of human probiotics.

In this review, we summarize the current knowledge on antibiotic resistance mechanisms in lactobacilli and bifidobacteria, as well as in other potential probiotic candidates, such as *Bacillus* strains. We did not consider enterococci because of the high prevalence of antibiotic resistance determinants in this genus and the obvious safety concerns.

## ANTIBIOTIC RESISTANCE DETERMINANTS IN *Lactobacillus*

The genus *Lactobacillus* is the largest group among the lactic acid bacteria (LAB) and likely the most widely used as a probiotic in a variety of foods, mainly meat and fermented dairy products. To date, 182 species have been described within the genus (list of prokaryotic names with standing in nomenclature; www.bacterio.cict.fr/), giving an idea of its complexity. With regard to antibiotic resistance, the vancomycin-resistant phenotype of some lactobacilli is perhaps the best characterized intrinsic resistance in LAB. Vancomycin comes into contact with the peptidoglycan precursors on the cell wall side of the cytoplasmic membrane and binds to the D-alanine/D-alanine terminus of the pentapeptide, preventing polymerization of peptidoglycan precursors. In several species of LAB, the terminal D-alanine residue is replaced by D-lactate or D-serine in the muramylpentapeptide, preventing vancomycin binding (Delcour et al., 1999) and therefore becoming resistant to the antibiotic. In addition, chromosomal mutations leading to antibiotic resistance phenotypes have also been described in lactobacilli. Flórez et al. (2007) identified a single mutation in the 23S rRNA gene reducing the affinity of erythromycin for the ribosome. This mutation conferred macrolide resistance in a strain of *L. rhamnosus*. In this respect, the transfer risk is considered to be very low for intrinsic resistance, or acquired resistance due to chromosomal mutation(s). Nevertheless, knowledge of the antibiotic resistance phenotypes may still be important, even in the absence of transferable resistance; since lactobacilli are commonly used in food and feed products, intrinsic resistance might still be relevant for the treatment of *Lactobacillus*-related bacteremia (Cannon et al., 2005).

Horizontally transferred antibiotic resistance genes, particularly those carried within mobile genetic elements, are the most likely to be transmitted and thus deserve particular attention. A major step in the differentiation between the intrinsic and the acquired antibiotic resistance in probiotic bacteria is the determination and the comparison of antibiotic susceptibility patterns of a representative number of different strains from each species. Although some effort has been made to this end, work has only been carried out for some antibiotics and particular *Lactobacillus* species. These include the most commonly used probiotic species such as *L. casei*, *L. acidophilus*, *L. reuteri*, or *L. rhamnosus*, among others, or the yogurt starter bacteria *L. delbrueckii* (Ammor et al., 2008b; Korhonen et al., 2008; Mayrhofer et al., 2010). However, given the taxonomic complexity of this microbial genus, there is still a lack of agreement on the resistance susceptibility breakpoints for most antibiotics. The use of molecular methods, such as microarray analysis and various PCR techniques is being extremely helpful in determining the genetic basis of the acquired resistance phenotypes. Moreover, the increasing availability of genome sequences and the cost reduction of genome sequencing facilities offer new possibilities for the screening of antimicrobial resistance genes (Bennedsen et al., 2011).

With regard to specific antibiotics, lactobacilli are usually sensitive to the cell wall-targeting penicillin and β-lactamase, but are more resistant to cephalosporins. As previously mentioned, many *Lactobacillus* species show a high level of resistance to vancomycin. Also, most inhibitors of nucleic acid synthesis seem to have a low inhibitory effect among the majority of *Lactobacillus* species. On the other hand, lactobacilli are generally susceptible to low concentrations of many inhibitors of protein synthesis, such as chloramphenicol, macrolides, lincosamides, and tetracycline, but their resistance to aminoglycosides is often high. Resistance to other antibiotics varies greatly among lactobacilli.

Several genes responsible for atypical antibiotic resistance properties among lactobacilli have been reported (**Table 1**). Chloramphenicol resistance genes (*cat*; chloramphenicol acetyltransferases) have been identified in *L. acidophilus*, *L. delbrueckii* subsp. *bulgaricus* (Hummel et al., 2007), and *L. johnsonii* (Mayrhofer et al., 2010) as well as in *L. reuteri* (Lin et al., 1996) and *L. plantarum* (Ahn et al., 1992). In addition, erythromycin resistance genes, responsible for the macrolides, lincosamides, and streptogramins (MLS) resistance phenotype, have been identified in several *Lactobacillus* species; the *erm*(B) gene, which encodes a rRNA methylase acting on the 23S ribosomal subunit, is the most frequently found of such genes, but others such as *erm*(A), *erm*(C), or *erm*(T) have also been detected (van Hoek et al., 2008c; Mayrhofer et al., 2010). The presence of genes coding for macrolide efflux pumps, such as *mef*(A), genes for lincosamide transferase [*lnu*(A); Cauwerts et al., 2006] and streptogramin A acetyltransferases [*vat*(E); Gfeller et al., 2003; Mayrhofer et al., 2010] have also been reported. However, the most common resistance determinants found in lactobacilli are the tetracycline resistance genes, which are sometimes found in combination (Ammor et al., 2008c). At least 11 different tetracycline resistance genes have been detected to date in lactobacilli, these include genes coding for ribosomal protection proteins [*tet*(W), *tet*(M), *tet*(S), *tet*(O), *tet*(Q), *tet*(36), *tet*(Z), *tet*(O/W/32/O/W/O), *tet*(W/O)] and efflux pumps [*tet*(K) and *tet*(L); Lahtinen et al., 2009]. Aminoglycoside resistance genes, such as *aac*(6′)-*aph*(2′′), *ant*(6), and *aph*(3′)-IIIa (Rojo-Bezares et al., 2006), and β-lactam resistance genes (*blaZ*) were found much less frequently in lactobacilli (Aquilanti et al., 2007).

**Table 1:** Antibiotic resistance determinants identified and characterized in lactobacilli, bifidobacteria, and probiotic *Bacillus* strains.

Gene(s)	Resistance	Mechanism	Location	Reference
***Lactobacillus***
*blaZ*	β-Lactams	Antibiotic hydrolysis	–	Aquilanti et al. (2007)
*vat*(E)	Quinupristin–dalfopristin	Antibiotic acetylation	–	Mayrhofer et al. (2010)
*Cat*	Chloramphenicol	Antibiotic acetylation	Plasmid	Ahn et al. (1992); Lin et al. (1996); Hummel et al. (2007); Mayrhofer et al. (2010)
*msrC*	MLS	Efflux	–	Thumu and Halami (2012)
*mef*(A)	Macrolide	Efflux	–	Cauwerts et al. (2006)
*aac*(6′)-*aph*(2′′), *ant*(6), *aph*(3′)-IIIa	Aminoglycoside	Enzymatic modification	–	Rojo-Bezares et al. (2006)
*erm*(B), *erm*(C), *erm*(T), *erm*(LF), *erm*(GT), *erm*(A)	MLS	Ribosomal methylation	Plasmid, transposon, chromosome	Tannock et al. (1994); Gfeller et al. (2003), Cauwerts et al. (2006); Aquilanti et al. (2007), Hummel et al. (2007); Klare et al. (2007), Ammor et al. (2008b); Egervärn et al. (2009), Mayrhofer et al. (2010); Thumu and Halami (2012)
*tet*(W), *tet*(M), *tet*(S), *tet*(O), *tet*(Q), *tet*(36), *tet*(Z), *tet*(W/O), *tet*(O/W/32/O/W/O)	Tetracycline	Ribosomal protection	Plasmid, transposon, chromosome	Aquilanti et al. (2007); Klare et al. (2007), Ammor et al. (2008b), van Hoek et al. (2008b,c)
*tet*(K)/*tet*(L)	Tetracycline	Efflux	Plasmid	Aquilanti et al. (2007); Ammor et al. (2008c), Devirgiliis et al. (2009); Thumu and Halami (2012)
***Bifidobacterium***
*erm*(X)	MLS	Ribosomal methylation	Transposon	van Hoek et al. (2008a)
*tet*(W), t*et*(M), *tet*(O), *tet*(W/32/O), *tet*(O/W)	Tetracycline	Ribosomal protection	Chromosome	Flórez et al. (2006); Kazimierczak et al. (2006), Ammor et al. (2008a); van Hoek et al. (2008b)
*tet*(L)	Tetracycline	Efflux	Chromosome	van Hoek et al. (2008b)
***Bacillus***
*aaD2*	Aminoglycoside	Antibiotic adenylation	Chromosome	Bozdogan et al. (2003)
*erm*(34)	MLS	Ribosomal protection	Chromosome	Bozdogan et al. (2004)
BCL-1	β-lactams	Antibiotic hydrolysis	Chromosome	Girlich et al. (2007)
*cat*(Bcl)	Chloramphenicol	Antibiotic acetylation	Chromosome	Galopin et al. (2009)

It is important to point out that many of the genetic determinants mentioned above are sometimes found in potentially mobile elements, such as transposons and plasmids, which could spread the antibiotic resistance genes mainly by conjugation mechanisms. The localization of these genes within the genome, their nucleotidic sequence, and the analysis of the flanking regions surrounding the antibiotic resistance genes may yield important clues as to the acquisition process of these determinants, and their source or origin (Aquilanti et al., 2007). Remarkably, some of these genes have been found to be transferred *in vitro* between strains of *Lactobacillus* and also from lactobacilli to different Gram-positive bacteria, including food pathogens, such as *Staphylococcus* (Tannock et al., 1994). On the other hand, lactobacilli may be able to acquire antibiotic determinants from other Gram-positive bacteria (Vescovo et al., 1983). In addition to *in vitro* studies, the potential risks associated with lactobacilli carrying transferable antibiotic resistance determinants have also been demonstrated in experimental animal models (Mater et al., 2008). The transfer of these determinants may be enhanced in the presence of antibiotic selective pressure (Feld et al., 2008). Taken together, these results support the hypothesis of the resistance gene reservoir within intestinal bacteria, and their role as traffickers in antibiotic resistance genes.

Another mechanism of resistance against antimicrobials known to be present in certain lactobacilli is that mediated by multidrug resistance (MDR) transporters. The role of drug resistance mediated by MDR systems in lactobacilli has been the subject of a previous review (Gueimonde et al., 2009). Although all lactobacilli have MDR homologs, information in this regard is limited, with only a few studies regarding the beer-spoiling *L. brevis* displaying resistance to hop compounds (Sakamoto et al., 2001), a proton motive force-dependent hop excretion transporter, named HorC, found in *L. lindneri* (Suzuki et al., 2005), or a MDR protein found to be involved in bile resistance in *L. reuteri* (Whitehead et al., 2008).

## ANTIBIOTIC RESISTANCE IN *Bifidobacterium* AND *tet*(W) UBIQUITY

Bifidobacteria are members of the Actinobacteria phylum, one of the main phylogenetic groups of the human gut microbiota (Sánchez et al., 2013). *Bifidobacterium* infections are extremely rare and have involved immunocompromised patients in a few cases (Ohishi et al., 2010; Barberis et al., 2012; Jenke et al., 2012), but, to the best of our knowledge, the five species of *Bifidobacterium* with QPS status (*B. adolescentis*, *B. animalis*, *B. bifidum*, *B. breve*, and *B. longum*; EFSA, 2012) have not been linked to any infective processes in healthy individuals. However, several strains displaying antibiotic resistance phenotypes have been characterized, and in many cases the phenotype has been linked to specific antibiotic resistance genes, representing a potential risk of transfer to other bacteria in the intestinal ecosystem (Ammor et al., 2008b).

Bifidobacteria are intrinsically resistant to mupirocin, an antibiotic that is being used in selective media for this genus. Mupirocin competes with isoleucine as a substrate for isoleucyl-tRNA synthetase, thus affecting protein synthesis. The resistance phenotype of bifidobacteria is a consequence of the synthesis of an atypical isoleucyl-tRNA synthetase that contains key amino acid residues responsible for the high level of mupirocin resistance (Serafini et al., 2011). Furthermore, they are not susceptible to high concentrations of aminoglycosides, most likely as a consequence of the lack of cytochrome-mediated drug transport (Mayrhofer et al., 2011). On the contrary, low concentrations of macrolides, vancomycin, chloramphenicol, beta-lactams, rifampicin, and spectinomycin, normally inhibit their growth (Zhou et al., 2005; Lahtinen et al., 2009). However, it is worth mentioning that a few streptomycin resistant strains have been characterized, leading to the conclusion that the resistance phenotype in these strains is due to chromosomal mutations, and not to the acquisition of specific antibiotic resistance genes, and therefore do not represent a potential risk of transferability. Thus, a high resistance to streptomycin was correlated with a mutation on the *rpsL* gene for ribosomal protein S12 in *B. bifidum* and *B. breve* (Kiwaki and Sato, 2009; Sato and Iino, 2010). Also, a *B. bifidum* strain displaying low erythromycin susceptibility was found to possess mutated 23S ribosomal RNA gene copies, likely to be responsible for the observed phenotype (Sato and Iino, 2010).

Data on antibiotic resistance determinants in bifidobacteria are relatively scarce, and are limited to tetracycline and macrolide antibiotics (**Table 1**). MDR proteins have been described in *B. longum* and *B. breve*. The transporters are able to confer low resistance levels to erythromycin, although their contribution to the MLS phenotype remains to be determined (Margolles et al., 2005; Price et al., 2006). Also, a gene coding for a ribosomal protection protein, *erm*(X), was identified in *B. animalis* subsp. *lactis* and *B. thermophilum*, as a part of the transposon Tn*5432* (van Hoek et al., 2008a).

Tetracycline resistance in *Bifidobacterium* deserves special attention. We have known for more than a decade, that proteins that protect the ribosome from the action of tetracyclines, the so-called *tet* genes, are commonly found in this genus (Scott et al., 2000; Gueimonde et al., 2010). The genes *tet*(W), *tet*(M), *tet*(O), *tet*(W/32/O), and *tet*(O/W) have been detected in several *Bifidobacterium* species, including *B. longum* (subsp. *infantis* and subsp. *longum*), *B. breve*, *B. animalis* subsp. *lactis*, *B. bifidum*, *B. pseudocatenulatum*, and *B. thermophilum* (Flórez et al., 2006; Kazimierczak et al., 2006; Aires et al., 2007, 2009; Ammor et al., 2008a; van Hoek et al., 2008b; Gueimonde et al., 2010). The gene *tet*(W) is especially ubiquitous; it has been detected at high frequencies in *B. longum* strains (Aires et al., 2007; Ammor et al., 2008a), and in all *B. animalis* subsp. *lactis* strains analyzed until now (Aires et al., 2007; Gueimonde et al., 2010). This last fact is very relevant, taking into account that *B. animalis* subsp. *lactis* strains are extensively used in the functional food industry, especially in fermented dairy products (Masco et al., 2005). The *tet*(W) gene in *Bifidobacterium* seems to be integrated in the chromosome and its surrounding regions vary depending on the strain, but very often the gene is flanked by transposase target sequences or genes coding for transposases, enzymes that catalyze the movement of DNA fragments between different locations by recognizing specific target sequences, suggesting that, under adequate conditions, the gene could be transferred (Kazimierczak et al., 2006; Ammor et al., 2008a; van Hoek et al., 2008a,b; Gueimonde et al., 2010). In fact, a *tet*(W) gene of *B. longum*, containing a transposase located within the conserved upstream region of the gene, and flanked by imperfect direct repeats, was transferable, at low frequencies, between *B. longum* and *B. adolescentis* under *in vitro* conditions (Kazimierczak et al., 2006). This suggests the potential of bifidobacteria to transfer antibiotic resistance genes to closely related bacteria. However, although attempts have been made to demonstrate the donor capacity of bifidobacteria to other enteric bacteria, to the best of our knowledge this has not been experimentally proved yet.

## ANTIBIOTIC RESISTANCE GENES IN OTHER PROBIOTIC STRAINS

Members of the genus *Bacillus* are aerobic or facultative aerobic, endospore-forming and rod-shaped Gram-positive bacteria, which inhabit a wide range of habitats, mostly soil and sediments. These bacteria do not belong to the commensal microbiota of the gastrointestinal tract, but some strains of the genus are included in food supplements and used in human nutrition as probiotics, notably *Bacillus clausii* (Ciffo, 1984). Furthermore, several *Bacillus* species have been employed for centuries in the manufacture of traditional, fermented dishes in Africa and Asia (Sarkar et al., 2002). Nowadays, certain *Bacillus* strains are used as feed additives, plant production products, biomass for animal feed, or enzyme/vitamin production (SCAN, 2000; Hong et al., 2005), and several species are included on the EFSA QPS list (EFSA, 2012). Many characteristics of probiotic *Bacillus* strains differ from those of other probiotic bacteria, including its ability to sporulate and the mechanisms of interaction with the human intestinal mucosa (Sánchez et al., 2009).

Regarding the presence of antibiotic-resistance mechanisms in *Bacillus*, macrolide-resistance genes present on extra-chromosomal elements have been identified in mobile elements, such as the plasmid-encoded *erm*(C) from *Bacillus subtilis* (Monod et al., 1986). Tetracycline resistance determinants have also been found in mobile elements, including the plasmid-encoded *tet*(L) gene from *Bacillus subtilis* (Phelan et al., 2011), and the *tet*(M) gene, contained within the conjugative transposon Tn*5397* of *Bacillus subtilis* (Roberts et al., 1999). Other tetracycline resistance genes, such as *tet*(K), have been observed in some *Bacillus* isolates (Neela et al., 2009). Recently, the presence of cfr-like genes in several *Bacillus* species has been reported. Cfr genes encode ribosome methyltransferases providing resistance to several classes of antibiotics including phenicols, oxazolidinone, lincosamides, pleuromutilins, and streptogramin A (Dai et al., 2010). However, these genes are not apparently expressed in the species assayed, in spite of being fully functional in other bacterial hosts (Hansen et al., 2012). In this regard, it is worth noting the presence of specific antibiotic resistance mechanisms in certain *Bacillus clausii* strains, which have been used as probiotics in humans, especially for the prevention of infectious bacterial diarrhea. For instance, the *erm*(34) gene has been identified in the probiotic *Bacillus clausii* DSM8716 strain (Bozdogan et al., 2004). Probiotic *Bacillus clausii* strains also harbor specific antibiotic-defense mechanisms, such as an aminoglycoside resistance gene (*aadD2*), a chloramphenicol acetyltransferase gene, *cat*(Bcl) or a β-lactamase (BCL-1; Bozdogan et al., 2003; Girlich et al., 2007; Galopin et al., 2009).

## CONCLUSION

Bacteria naturally present in foods or food supplements, or deliberately added to them, including probiotic bacteria, constitute a potential source of antibiotic resistance determinants. Especially some fermented foods, such as dairy products, possess an extremely high bacterial density, mostly composed of LAB, quantitatively comparable with the microbial population found in some parts of the human intestine. This microbial population represents a huge reservoir of antibiotic resistance genes whose ingestion could influence the presence, establishment, and dynamics of antibiotic resistance bacteria in our body.

## Conflict of Interest Statement

The authors declare that the research was conducted in the absence of any commercial or financial relationships that could be construed as a potential conflict of interest.
